# DEX, Delirium and Dilemma

**DOI:** 10.21470/1678-9741-2022-0002

**Published:** 2023

**Authors:** Rohan Magoon, Shalvi Mahajan, Jes Jose

**Affiliations:** 1 Department of Cardiac Anaesthesia, Atal Bihari Vajpayee Institute of Medical Sciences (ABVIMS) and Dr. Ram Manohar Lohia Hospital, New Delhi, India; 2 Department of Anaesthesia and Intensive Care, Post Graduate Institute of Medical Education & Research (PGIMER), Chandigarh, India; 3 Department of Cardiac Anesthesiology, Sri Jayadeva Institute of Cardiovascular Sciences and Research, Jayanagar, Bengaluru, Karnataka, India

**Keywords:** Cardiac Surgery, Dexmedetomidine, Meta-Analysis, Delirium, Randomized Controlled Trials.

## Abstract

Dexmedetomidine has been subjected to an extensive evaluation for its’ role in the prevention of postoperative delirium following cardiac surgery. In striking contrast to the preexisting meta-analysis supporting postoperative delirium-reduction with dexmedetomidine, few recently concluded multicentric large scale randomized controlled trials suggest otherwise. This article aims to present a nuanced perspective of the evolving controversy by attempting to decode the apparent incongruences in the literature accumulating off-late, which is particularly pertinent amidst an ever-escalating heterogeneity in the current research ecosystem.

Ever since the first report of postoperative delirium (POD) following cardiac surgery in 1964, it remains an ardently researched domain^[^1^-^4^]^. This is principally attributed to the fact that the incidence of POD continues to range between 13 and 50% despite major advancements in cardiac surgical conduct^[^2^,^3^]^. The POD-associated morbidity-mortality, caregiver suffering, and the healthcare burden buttress the importance of a sound prediction and prevention strategy^[^1^-^4^]^.

Talking of the pharmacological prevention of POD, dexmedetomidine (DEX) has been subjected to an extensive evaluation with mixed results in the available literature^[^4^,^5^-^8^]^. While a number of existing meta-analyses support POD-reduction with DEX in a cardiac surgical setting^[^5^,^6^]^, few large scale randomized controlled trials (RCTs) conducted off-late suggest otherwise^[^7^,^8^]^. In this context, “Dexmedetomidine for reduction of atrial fibrillation and delirium after cardiac surgery (DECADE)” - the placebo-controlled RCT by Turan et al.^[^8^]^ contemplated across six academic hospitals in the United State of America and later published in The Lancet in the year 2020 - captivates attention. The DECADE trial studying atrial fibrillation and POD co-primarily randomized 798 patients to receive either DEX or saline infusion at 0.1 µg/kg/hour prior to incision, escalated to 0.2 µg/kg/hour at the end of bypass and to 0.4 µg/kg/hour postoperatively till 24 hours. Following the analysis of data of 794 patients, the trial failed to outline an amelioration of either of the unfavorable outcomes, rather demonstrated a non-significant worsening of POD (17% and 12% in the DEX and placebo groups, respectively)^[^8^]^. This portrays a striking contradiction to the findings of the previous meta-analysis of the decade, quite literally^[^5^,^6^,^8^]^.

Premised on the evolving controversy, an updated meta-analysis was contemplated by Li et al.^[^9^]^ comprising of a total of 2,813 adult patients across 15 RCTs (each featuring comparative DEX and placebo-control/other anesthetic-control groups and POD as a primary/secondary outcome) which also included patients of the DECADE trial^[^8^]^. The pooled results emanating from this very recent meta-analysis published in 2021 revealed that DEX could significantly attenuate the risk of POD in comparison to the controls, with an odds ratio (OR) of 0.56, 95% confidence interval (CI) of 0.36-0.89, *P*-value = 0.0004, and 64% I2 coefficient^[^9^]^. Ahead of the demonstration of 44% POD risk reduction with DEX, the total POD-incidence funnel plot did not connote a significant publication bias in the meta-analysis. The additional findings of the subgroup analysis are outlined in Table 1^[^9^]^.

**Table 1:** Findings of the subgroup analysis of the Li et al. meta-analysis^[^9^]^.

• Stratified by age:
DEX reduced POD incidence in adults < 65 years of age and not in those ≥ 65 years.^*^
• Stratified by the timing of DEX administration:
Postoperative use of DEX decreased POD incidence while an intraoperative use didn’t.
• Stratified by the control group:
DEX favored POD prevention with other sedatives as controls in contrast to the placebo controls.^**^

DEX=dexmedetomidine; POD=postoperative delirium

^*^Age, by itself, entails an enhanced risk to the development of POD^[^10^]^

^**^A significant contribution from the Dexmedetomidine for reduction of atrial fibrillation and delirium after cardiac surgery (DECADE) trial in the placebo control data could have compounded this subgroup analysis finding, the additional concerns regarding the interpretation of the DECADE trail results have also been discussed in the following text of the manuscript^[^9^,^10^]^

Although the primary conclusions of the DECADE trial and Li et al.^[^9^]^ meta-analysis appear incongruous at first sight, a closer look resolves the dilemma to an extent^[^8^]^. As described previously, the DECADE trial initiated DEX infusion intraoperatively wherein the absence of an eventual POD reduction is actually consistent with the Li et al.^[^9^]^ subgroup analysis, which also limits the description of POD-reduction potential to a postoperative drug administration (Table 1)^[^8^,^9^]^. Li et al.^[^9^]^ speculate intraoperative hypotension (IOH) as a likely mechanism of lack of POD protection of intraoperative DEX and elaborate that the former was not recorded in many of the included RCTs^[^9^]^. Turan et al.^[^8^]^ also implicate IOH for the non-significant rise in POD in the DECADE trial given the incidence of the same was 57% and 36% in the DEX and placebo groups, respectively. However, no significant association between IOH and POD materialized in the Wang et al.^[^10^]^ post-hoc analysis of the DECADE trial including data from three Cleveland Clinic hospitals, outlining an adjusted OR of 0.94 (95% CI: 0.81-1.09; *P*-value = 0.419) for a doubling in the area under the curve of mean arterial pressure < 60 mmHg, after applying thorough logistic regression models and adjusting for 11 confounders.

As far as POD reduction with postoperative DEX is concerned, an enlightening discussion transpired in a leading cardiothoracic anesthesia journal, the Journal of Cardiothoracic and Vascular Anesthesia^[^11^,^12^]^. An eleven-RCT meta-analysis by Abowali et al.^[^11^]^ concluded an absence of a statistically meaningful POD reduction with postoperative DEX in comparison to propofol sedation. Nonetheless, Heybati et al.^[^12^]^ highlighted that Abowali et al.^[^11^]^ managed to erroneously include two RCTs, one administering DEX intraoperatively with another employing DEX as an adjunct to propofol. Subsequently, Heybati et al.^[^12^]^ reanalyzed the data of the remaining nine RCTs only to discover a significant POD reduction with postoperative DEX (random-effects OR of 0.18, 95% CI: 0.05-0.65, with an I2 = 52% denoting a moderate heterogeneity level). Needless to say, the readjusted results were in agreement with the recent Li et al.^[^9^]^ meta-analysis.

Looking beyond the categorical details of the meta-analysis^[^13^]^, there are subtle intricacies of the individual DEX-delirium RCTs which necessitate close consideration^[^9^,^14^-^17^]^. Table 2 enlists some of the pertinent points to assess while interpreting the results of these RCTs^[^9^,^14^-^17^]^. For instance, the POD protective role evaluation of DEX in the Turan et al. DECADE trial may have been compounded by the lack of a protocolized anesthetic regime, titration of the study drug, and other sedatives administration at the discretion of the anesthesiologist and disregard to the group-specific cumulative sedative drug dosages being utilized^[^8^,^18^,^19^]^. At the same time, exempting a formal preoperative cognitive status assessment can pose important concerns as adequate literature exists to suggest preoperative anxiety-depression and low mini-mental state examination score as independent risk-factors for POD following cardiac surgery^[^14^-^17^]^. Notably, the Li et al.^[^9^]^ meta-analysis excluded the RCTs which did not rule out a preoperative cognitive impairment.

**Table 2:** Intricacies of the DEX-delirium RCTs to be considered while interpreting the results^[^9^,^14^-^17^]^.

• Whether or not preoperative cognitive impairment has been ruled out by a mini-mental state examination or other objective tools.
• If POD classifies as the primary or the secondary outcome.
• Sample-size involved and the approach to randomization-allocation-masking.
• The specified method of delirium assessment (CAM, CAM-ICU, DSM-IV, DSM-V, ICDSC, RASS).
• The timing and dosage protocol of DEX and other sedative agents.
• Nature of the control group assigned.
• Miscellaneous factors like anesthetic regime and depth, type of surgical procedure, perioperative management, cerebral perfusion technique, lowest Hb-MAP-temperature, glucose-homeostasis, blood-transfusion, oxygenation-perfusion strategy, operative-ACC-CPB times, etc.

ACC=aortic cross-clamping; CAM=Confusion Assessment Method; CAM-ICU=Confusion Assessment Method for the intensive care unit; CPB=cardiopulmonary bypass; DEX=dexmedetomidine; DSM-IV=Diagnostic and Statistical Manual of Mental Disorders, Fourth Edition; DSM-V=Diagnostic and Statistical Manual of Mental Disorders, Fifth Edition; Hb=hemoglobin; ICDSC=Intensive Care Delirium Screening Checklist; MAP=mean arterial pressure; POD=postoperative delirium; RASS=Richmond Agitation Sedation Scale; RCTs=randomized controlled trials

Even from a holistic research standpoint, the traditionally cited evidence hierarchy of meta-analysis over and above multiple and single RCTs is an oversimplistic rendition, mandating a cautious interpretation^[^13^]^. Amidst an ongoing surge in the number of meta-analysis, it becomes imperative to realize that meta-analysis of small RCTs can be conducive to an overestimation of treatment effects, often reformed by larger RCTs which follow^[^13^]^. In conclusion, large scale multicentric RCTs and meta-analysis of varying RCT counts coexist in the present heterogeneous research ecosystem wherein a superficial perusal of the conclusions can be misleading, particularly with the methodological issues, potential biases, and rhetorical presentation techniques being an inexorable part of the vignette^[^13^,^20^]^.

## References

[1] Egerton N, Kay JH. (1964). Psychological disturbances associated with open heart
surgery. Br J Psychiatry.

[2] Kumar AK, Jayant A, Arya VK, Magoon R, Sharma R. (2017). Delirium after cardiac surgery: a pilot study from a single
tertiary referral center. Ann Card Anaesth.

[3] Brown CH. (2014). Delirium in the cardiac surgical ICU. Curr Opin Anaesthesiol.

[4] Magoon R, Kumar AK, Malik V, Makhija N. (2020). Dexmedetomidine and postoperative delirium: decoding the
evidence!. J Anaesthesiol Clin Pharmacol.

[5] Wu M, Liang Y, Dai Z, Wang S. (2018). Perioperative dexmedetomidine reduces delirium after cardiac
surgery: a meta-analysis of randomized controlled trials. J Clin Anesth.

[6] Wang G, Niu J, Li Z, Lv H, Cai H. (2018). The efficacy and safety of dexmedetomidine in cardiac surgery
patients: a systematic review and meta-analysis. PLoS One.

[7] Subramaniam B, Shankar P, Shaefi S, Mueller A, O'Gara B, Banner-Goodspeed V (2019). Effect of intravenous acetaminophen vs placebo combined with
propofol or dexmedetomidine on postoperative delirium among older patients
following cardiac surgery: the DEXACET randomized clinical
trial. JAMA.

[8] Turan A, Duncan A, Leung S, Karimi N, Fang J, Mao G (2020). Dexmedetomidine for reduction of atrial fibrillation and delirium
after cardiac surgery (DECADE): a randomised placebo-controlled
trial. Lancet.

[9] Li P, Li LX, Zhao ZZ, Xie J, Zhu CL, Deng XM (2021). Dexmedetomidine reduces the incidence of postoperative delirium
after cardiac surgery: a meta-analysis of randomized controlled
trials. BMC Anesthesiol.

[10] Wang J, Mao G, Malackany N, Marciniak D, Donaldson C, Wakefield B (2022). Association between perioperative hypotension and postoperative
delirium and atrial fibrillation after cardiac surgery: a post-hoc analysis
of the DECADE trial. J Clin Anesth.

[11] Abowali HA, Paganini M, Enten G, Elbadawi A, Camporesi EM. (2021). Critical review and meta-analysis of postoperative sedation after
adult cardiac surgery: dexmedetomidine versus propofol. J Cardiothorac Vasc Anesth.

[12] Heybati K, Deng J, Zhou F, Ali S. (2022). Current evidence demonstrates a significant reduction in the
incidence of delirium with postoperative dexmedetomidine versus propofol
sedation. J Cardiothorac Vasc Anesth.

[13] Bartels K, Sessler DI. (2022). Meta-analyses of clinical trials: are we getting lemonade from
lemons?. Br J Anaesth.

[14] Chen H, Mo L, Hu H, Ou Y, Luo J. (2021). Risk factors of postoperative delirium after cardiac surgery: a
meta-analysis. J Cardiothorac Surg.

[15] Li CW, Xue FS, Hu B. (2021). Determining association between blood glucose variability and
postoperative delirium in acute aortic dissection patients: methodological
issues. J Cardiothorac Surg.

[16] Guenther U, Theuerkauf N, Frommann I, Brimmers K, Malik R, Stori S (2013). Predisposing and precipitating factors of delirium after cardiac
surgery: a prospective observational cohort study. Ann Surg.

[17] Humphreys JM, Denson LA, Baker RA, Tully PJ. (2016). The importance of depression and alcohol use in coronary artery
bypass graft surgery patients: risk factors for delirium and poorer quality
of life. J Geriatr Cardiol.

[18] Magoon R. (2020). Implications of practice variability: comment. Anesthesiology.

[19] Magoon R. (2020). Precision cardiac anesthesia: welcome aboard!. J Cardiothorac Vasc Anesth.

[20] Magoon R, Jose J. (2020). Safeguarding anaesthesia research from spin. Br J Anaesth.

